# Therapeutic potential of chrysin nanoparticle-mediation inhibition of succinate dehydrogenase and ubiquinone oxidoreductase in pancreatic and lung adenocarcinoma

**DOI:** 10.1186/s40001-022-00803-y

**Published:** 2022-09-08

**Authors:** Eman M. Ragab, Doaa M. El Gamal, Tarek M. Mohamed, Abeer A. Khamis

**Affiliations:** Biochemistry Division, Chemistry Department, Faculty of Science, Tanta University, Tanta, 31527 Egypt

**Keywords:** Cancer, Mitochondria, Chitosan, Chrysin, Succinate dehydrogenase

## Abstract

Pancreatic adenocarcinoma (PDAC) and lung cancer are expected to represent the most common cancer types worldwide until 2030. Under typical conditions, mitochondria provide the bulk of the energy needed to sustain cell life. For that inhibition of mitochondrial complex ΙΙ (CΙΙ) and ubiquinone oxidoreductase with natural treatments may represent a promising cancer treatment option. A naturally occurring flavonoid with biological anti-cancer effects is chyrsin. Due to their improved bioavailability, penetrative power, and efficacy, chitosan–chrysin nano-formulations (CCNPs) are being used in medicine with increasing frequency. Chitosan (cs) is also regarded as a highly versatile and adaptable polymer. The cationic properties of Cs, together with its biodegradability, high adsorption capacity, biocompatibility, effect on permeability, ability to form films, and adhesive properties, are advantages. In addition, Cs is thought to be both safe and economical. CCNPs may indeed be therapeutic candidates in the treatment of pancreatic adenocarcinoma (PDAC) and lung cancer by blocking succinate ubiquinone oxidoreductase.

## Background

Cancer remains a leading cause of death worldwide, with more than 18 million new cases diagnosed [1]. During the 1990s, rapid scientific progress in cancer led to the development of new diagnostic techniques and revolutionary targeted medicines. Epidemiological measures of pancreatic cancer, including incidence, prevalence, and mortality, vary widely throughout the African continent. Gender was not specified in 1188 patients with 355,317 new cases predicted before 2040 [2]. In Egypt, lung cancer (LC) represents approximately 4.6% of all cancers in both sexes. Deaths from LC in Egypt reached 4,429 per 100,000 of the population in 2002 [3]. The most popular cancer forms, pancreatic and LC, are expected to have the highest prevalence and death rate in the United States until 2030 [4].

## Lung cancer

**Fig. 1:**
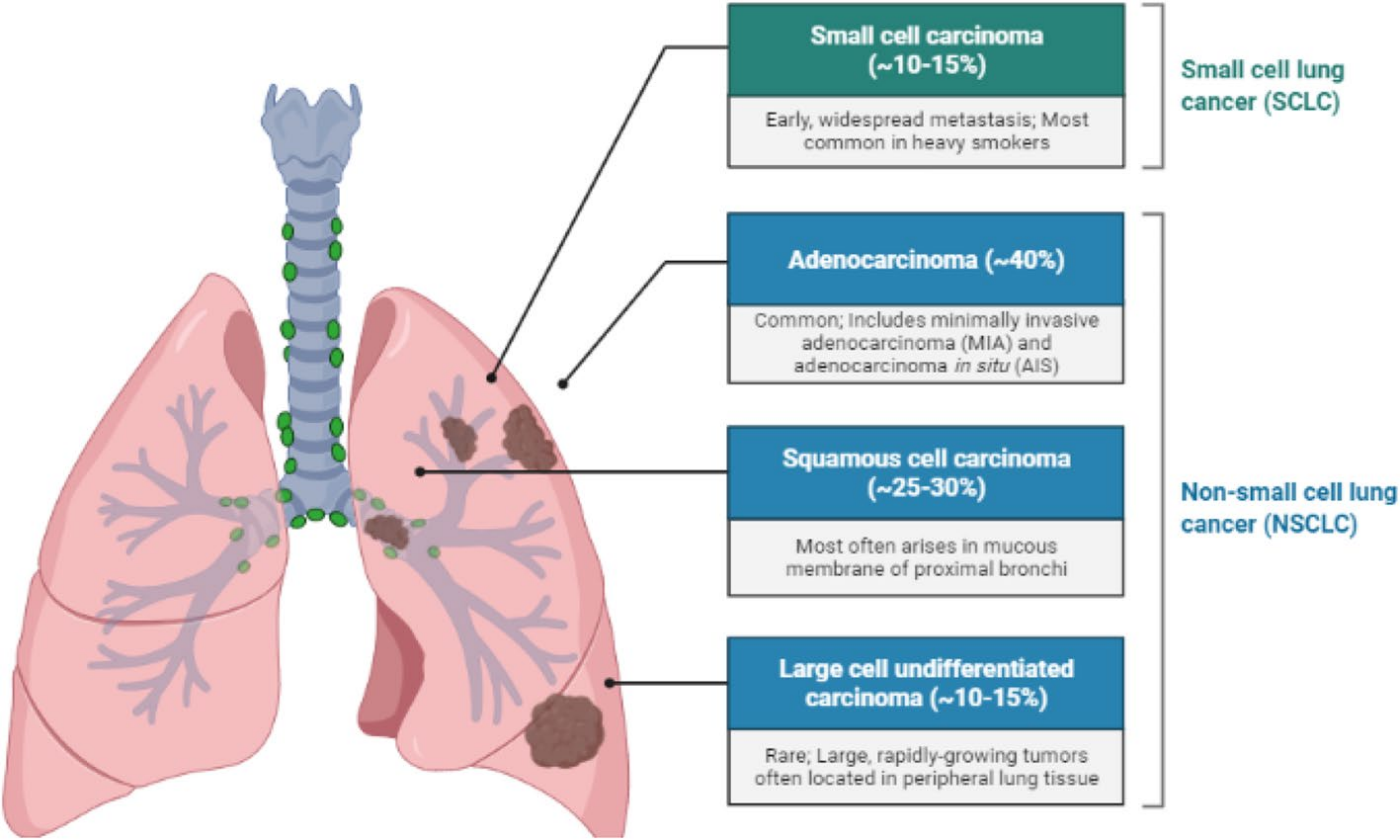
Most typical forms of lung cancer

Lung cancer (LC) is the main cause of death worldwide, with more than 1.8 million people dying from LC each year. LC is conventionally divided into two types: small cell lung cancers (SCLC; 15% of LC cases) and non-small cell lung cancers (NSCLC, 85% of LC cases; Fig. 1). The 5-year relative survival rate for LC is extremely low (16.8%), as approximately 80% of LC patients are discovered at a progressed stage that is rarely responsive to treatment with current therapies [5]. LC is associated with a higher symptom burden than other cancers. A previous qualitative study reported that dyspnea played a primary role in the onset of fatigue in patients with LC. Pain in patients with LC may be caused by the tumor itself, treatment, diagnostic tests, surgeries, procedures (e.g., biopsy, puncture, mediastinoscopy, thoracotomy), local or metastatic invasion of a tumor into the chest with consequent inflammation or pulmonary embolism of the invaded area, or due to tumoral compression of bones, nerves, or other organs [6]**.** Although there are numerous signaling pathways, including the MAPK, PI3K, oxidative stress response, RNA splicing, processing, and nucleosome remodeling pathways, have been linked to causal gene changes [7]**.** However, the anaerobic pathway and the oxidative pathway are the two primary pathways used by cancer cells to transfer energy. The former includes glycolysis, which is connected to the Warburg effect and lactate generation. The oxidative route, which can also involve glycolysis but necessitates mitochondrial oxygen consumption, yields more ATP from a wider range of substrates [8]. Metabolic studies on 9 individuals with varied forms of lung cancer showed metabolic variability between tumours. They also showed that oxidative lung cancers depend on a different carbon source than glucose to feed the TCA cycle given that only 8% of the acetyl-CoA originates from [13C]-glucose carbons [9]**.** There is a critical need for novel diagnostic and therapeutic agents to decrease death related to lung cancer and increase patient survival [10]. Regardless of developments in diagnosis and treatment, only 10.9% of individuals with LC live 5 years or more. The 5-year relative survival rates for LC only increased by 1% (18% to 19%) from 2004 to 2014 in the United States [11] and by 8.5% (19.7% to 28.2%) from 2006 to 2012 in South Korea. We demonstrated the validity of inhibiting the mitochondrial trifunctional enzyme complex in oxidative lung tumors.

## Pancreatic adenocarcinoma

Pancreatic adenocarcinoma (PADC) is malignant epithelial neoplasia with glandular (ductal) differentiation that originates from the exocrine part of the pancreas and primarily originates in the head of the pancreas. The causes of pancreatic cancer have yet to be fully elucidated, although risk factors have been identified including cigarette smoking, positive family history, genetics, diabetes mellitus, obesity, dietary factors, alcohol use, and physical inactivity [12]**.**

Pancreatic cancer is an intractable malignancy and is the seventh leading cause of global cancer deaths in industrialized countries and the third most common in the USA [13]. Pancreatic cancer will surpass colorectal and breast cancer as the second most common cause of cancer-related deaths in Germany by 2030 [14]**.** Based on GLOBOCAN 2018 estimates, pancreatic cancer is the 11^th^ most common cancer in the world accounting for 458,918 new cases and causing 432,242 deaths (4.5% of all deaths caused by cancer) in 2018 [15]. The first systematic review of pancreatic cancer in the African continent included a total of 9259 patients. Most articles were cohort studies that included data on epidemiology. The majority of publications were from the 2000s to the present day. There exists a disparity in pancreatic cancer publication volume, with a majority of studies from two countries, Egypt and Nigeria. Further research is needed to determine country-level factors that may account for this disparity [2].

### Mechanism of disease

**Fig. 2:**
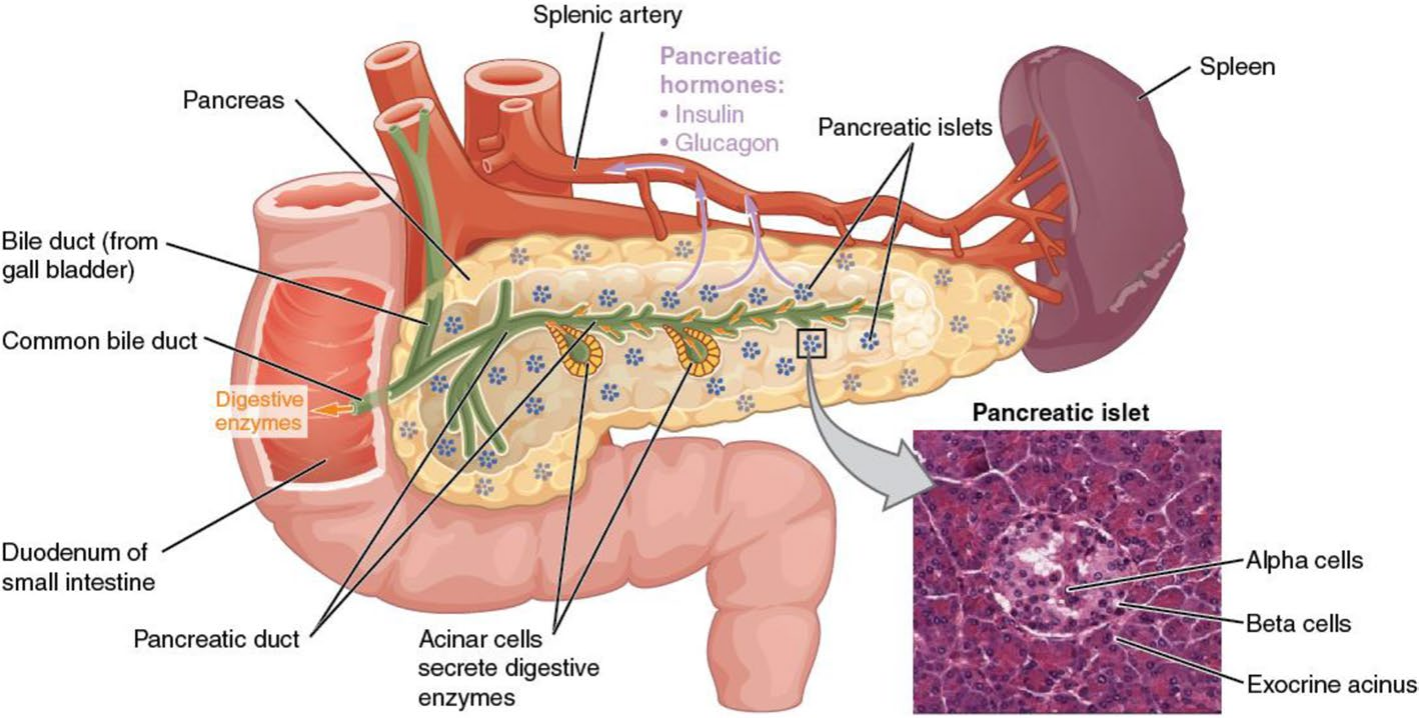
Anatomy and physiology of the pancreas [81]

The pancreas is comprised of separate functional units which regulate two important physiological processes, digestion and glucose metabolism. Acinar and duct cells from the exocrine pancreas. Acinar cells, which produce digestive enzymes, are the main constituents of pancreatic tissue. Acinar cells are grouped into grape-like clusters at the smallest termini of the branching pancreatic duct system. Pancreatic ducts, which add mucous and bicarbonate to enzymatic secretions, form a network with increasing luminal width as main and accessory pancreatic ducts reach the duodenum. The endocrine pancreas secretes hormones into the bloodstream using four specialized cell types arranged into compact islets embedded inside acinar tissue. The synthesis of glucagon and insulin by α- and β-cells, respectively, regulates the use of glucose. The secretory capabilities of the other pancreatic cell types are modulated by pancreatic polypeptide and somatostatin, which are produced in PP and δ-cells, respectively (Fig. 2) [16].

PADC is associated with one of the bleakest prognoses in all of medicine. PADC commonly goes undetected during early stages due to a lack of distinct symptoms and limitations in diagnostic techniques. PDAC is characterized by a hypoxic microenvironment and extensive stroma [17]. Pancreatic stellate cells (PSC) are crucial profibrogenic cells in the stromal reaction around tumor cells and are thought to be necessary for normal pancreatic architecture [18]. PSCs change into myofibroblast-like cells in response to tissue hypoxia, inflammation, or damage [19]. Smooth muscle actin is expressed by activated PSCs, and extracellular matrix components (ECM) such as collagen, fibronectin, and laminin are produced in excess [20]**.** The equilibrium between ECM production and breakdown is disrupted in cancer, eventually leading to a thick, fibrotic stroma [19]. Resulting in fibrotic tissue compressing vessels, thereby restricting tumor vascularization and exacerbating tissue hypoxia [21]**.** Processes medicated by hypoxia-inducible factor 1α (HIF-1α), such as epithelial-to-mesenchymal transition (EMT), are triggered by hypoxic environments [22]. EMT enhances tumor cell proliferation, metastatic dissemination, and chemo- and radio-resistance, ultimately leading to tumor progression and recurrence in PDAC patients. As a result, addressing mitochondrial metabolism, namely, redox metabolism, represents a promising therapeutic strategy.

## Mitochondria

Mitochondria are organelles that function in cellular respiration, specifically connecting the reduction of molecular oxygen (O_2_) to the synthesis of ATP. Mitochondria are the site of several critical metabolic pathways, including the citric acid cycle (CAC), oxidation, the urea cycle, and heme production. Mitochondria have specialized transporters that facilitate the movement of metabolites into and out of cells [23]. Accordingly, the overall function of mitochondria is intertwined with the demands and functions of their cellular environment. Given the essential function of mitochondria in metabolism, altered mitochondrial metabolism is evident in a variety of physiological and pathological states. Indeed, metabolic signs of mitochondrial abnormalities are beginning to emerge in aging and neurodegeneration, [24] as well as in cancer [25]. These findings are of interest for the diagnosis and treatment of mitochondrial diseases.

### Tricarboxylic acid cycle

**Fig. 3:**
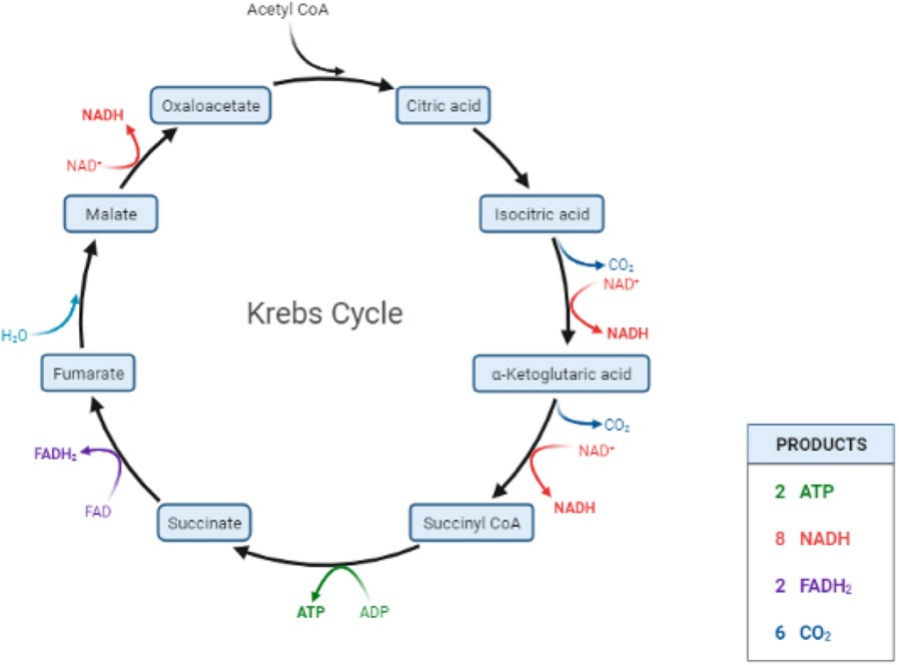
Overview of the TCA cycle

The tricarboxylic acid cycle (TCA), also known as the CAC or Krebs cycle, is an amphibolic process that operates in mitochondria and has evolved as a prominent metabolic center in most eukaryotic species [26]. Importantly, any fuel molecule, such as carbohydrates, lipids, or amino acids, that can be converted into acetyl-CoA or a TCA cycle intermediary can enter aerobic metabolism through this cycle. The TCA cycle's main role is to completely oxidize acetyl-CoA to CO_2_ and regenerate oxaloacetate [27]. This is the result of a set of eight chemical reactions (Fig. 3). The TCA is regarded to be the primary mechanism in energy metabolism as it is required for the production of the high-transfer-potential electron carriers nicotinamide adenine dinucleotide (NADH H^+^) and flavin adenine dinucleotide, (FADH_2_) [28]**.** NADH H^+^ and FADH_2_ are key shuttles for the passage of high-energy electrons to the terminal electron acceptor O_2_ via an array of electron carriers known as the electron transport chain (ETC). The ETC's major function is to create an electrochemical gradient across the mitochondrial inner membrane (IMM), which is essential for chemiosmotic ATP synthesis (OXPHOS) [29]**.** Importantly, the TCA cycle serves as a source of precursors for many other biological compounds, including non-essential amino acids, nucleotide bases, and porphyrin [27].

### Mitochondrial respiratory chain

**Fig. 4:**
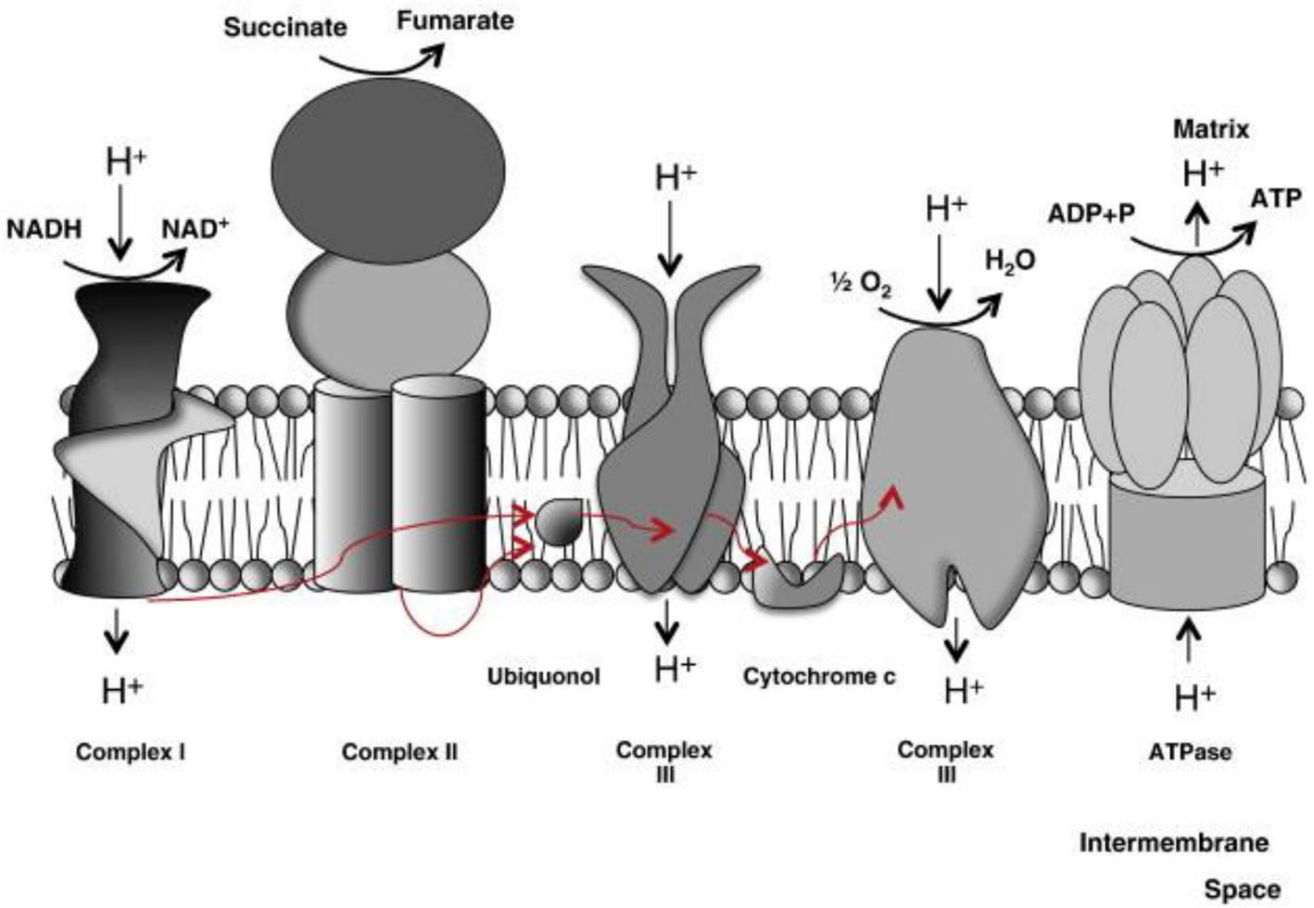
Electron transport chain [82]

The ETC is made up of five multimeric protein complexes that are found in the inner mitochondrial membrane (Fig. 4). Complex I contains approximately 46 subunits of reduced nicotinamide adenine dinucleotide (NADH) dehydrogenase–ubiquinone oxidoreductase, succinate dehydrogenase–ubiquinone oxidoreductase (complex II (CΙΙ), 4 subunits), ubiquinone–cytochrome c oxidoreductase (complex III, 11 subunits), and ATP synthase (complex V, approximately 16 subunits). Ubiquinone (coenzyme Q10) and cytochrome c (Cyt c) are two tiny electron carriers required by the respiratory chain. Two steps are involved in ATP production. First, electrons (hydrogen ions produced from NADH and reduced flavin adenine dinucleotide in intermediate metabolism) are transferred to molecular oxygen with the complexes, resulting in the production of water. Complexes I, III, and IV pump protons across the mitochondrial inner membrane (i.e., from the matrix to the intermembrane gap) at the same time. Complex V (ATP synthase), the world's tiniest rotary motor, generates ATP by returning these protons to the mitochondrial matrix [30]**.** Mitochondria are the only organelles in the cell, other than the nucleus, that contain DNA (termed mt DNA) and machinery for RNA and protein synthesis. Each cell has hundreds or thousands of mitochondria, each with approximately five mitochondrial genomes. As mt DNA has only 37 genes, nuclear DNA (nDNA) encodes the majority of the 900 mitochondrial gene products, which are imported from the cytoplasm [31].

#### Succinate dehydrogenase

**Fig. 5:**
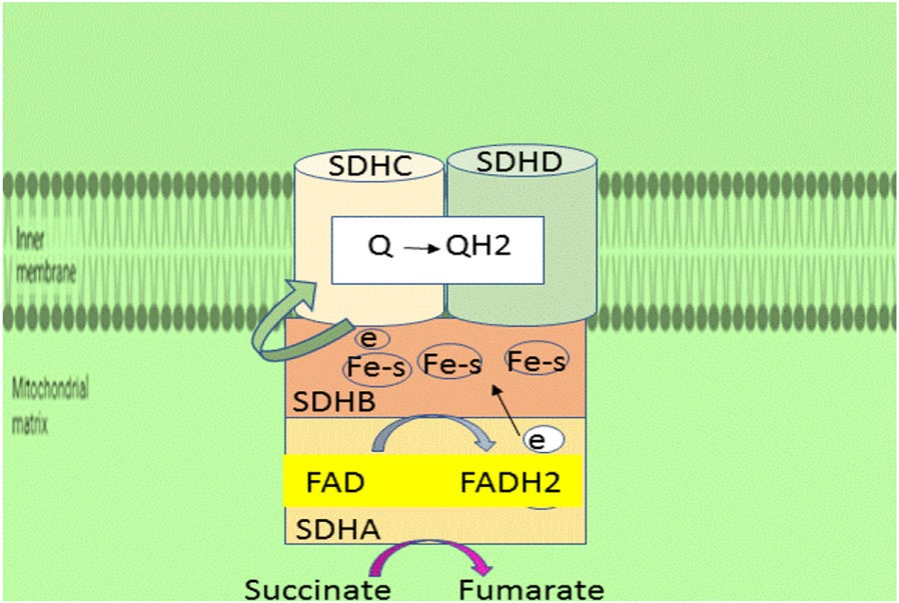
SDH complex (simplified). The flavin cofactor (FAD) is found in the catalytic subunit SDH subunit A, which takes electrons from succinate and transmits them to the Fe–S center in the SDH subunit B subunit. The electrons are subsequently transported through the SDHC and SDHD subunits' embedded ubiquinone pool. Within the mitochondrial inner membrane space, reduced Q (QH2 = ubiquinol) delivers electrons to complex III

**Fig. 6:**
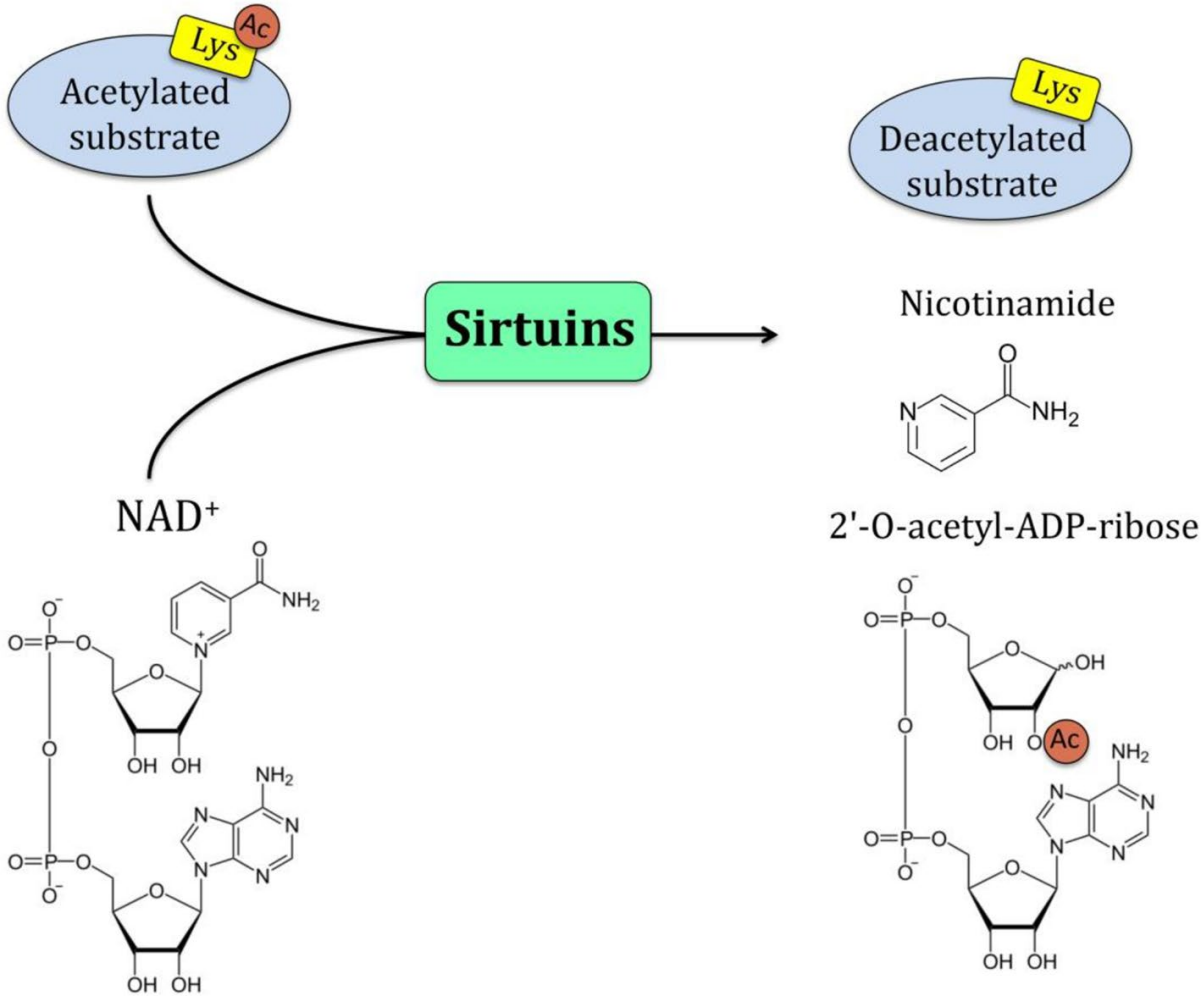
Deacetylation Mechanism of SIRT3 by nicotinamide adenine dinucleotide on several Lys residues in SDHA of mitochondria Complex ΙΙ [83]

Succinate dehydrogenase (SDH), also known as succinate Coenzyme Q oxidoreductase (SQR)**,** is a mitochondrial heterotetrameric enzyme that functions as CIΙ in the TCA cycle and the mitochondrial electron transport chain [32]. SDH catalyzes the TCA cycle's oxidation of succinate to fumarate and sends electrons to the respiratory chain's ubiquinone (coenzyme Q) pool. SDH in eukaryotes is made up of four subunits that are encoded by the nuclear genome. SDH is the only oxidative phosphorylation complex that lacks mitochondrial genome-encoded components and the only respiratory complex to not pump protons across the inner membrane (IM) during its catalytic cycle [27]. The SDH has a hydrophilic head that protrudes into the matrix compartment and a hydrophobic tail that is entrenched within the IM and has a brief segment that protrudes into the soluble intermembrane region (IMS). The catalytic subunits in the hydrophilic head that protrude into the mitochondrial matrix are SDH subunit A (SDHA) and subunit B (SDHB). The ubiquinone-binding and membrane-anchoring components of SDH are SDHC and SDHD, respectively (Fig. 5). Flavination of SDHA, which is required for the formation of the SDH complex, is required for the formation of the primary catalytic subunit in the conversion of succinate to fumarate in SDHA which contains a succinate binding site. The SDHB protein mediates electron transport to the ubiquinone pool and contains three Fe–S centers. SDHC and SDHD bind ubiquinone, which causes protons to be produced, thereby leading to the creation of ATP. SDH catalyzes the oxidation of succinate to fumarate, as shown in Fig. 5. In this equation, two hydrogen atoms are removed from succinate by flavin adenine dinucleotide (FAD), a prosthetic group that is tightly attached to SDH. Two electrons from the reduced SDH–FADH2 complex are then transferred to ubiquinone (Q), a soluble component of the electron transport system complex II. Ubiquinone is then reduced to ubiquinol (QH2) [33]. Regulation of SDH The catalytic activity of SDH is modulated by post-translational phosphorylation and acetylation in addition to suppression of active sites. [33]. Sirtuin-3 (SIRT-3) is the major NAD-dependent protein deacetylase. predominantly localized in the mitochondrial matrix. SIRT-3 catalyzes the oxidation of succinate to fumarate and is a primary physiological regulator of SDH activity. Reversible acetylation of several Lys residues in SDHA has been demonstrated to lead to reduced SDHA enzymatic activity, thereby lowering SDH activity and increasing succinate levels [34]. At high levels of reduced cofactors such as NADH H^+^ and FADH_2_ present in the mitochondria, there is no requirement for more oxidation of acetyl-CoA in the Krebs cycle for making of these cofactors to support OXPHOS. Thus, it would be sensible to propose that acetylation of SDHA merely decreases the Krebs cycle, as this process will also result in gathering, of acetyl-CoA in the mitochondria. On the other hand, when NAD levels increase in the mitochondria, SIRT3 and other NAD-dependent deacetylases are activated and deacetylate SDHA and other acetylated components of the Krebs cycle. In agreement with stimulation of catalytic activities of metabolic enzymes, such as glutamate dehydrogenase and acetyl-CoA synthetase 2 by deacetylation, deacetylation of SDH A also stimulates complex II or succinate dehydrogenase activity to promote the Krebs cycle for the generation of reduced NADHH^+^ and FADH_2_, as they are the electron donors for ATP synthesis in OXPHOS (Fig. 6) [33, 35].

## SDH deficiency

**Fig. 7:**
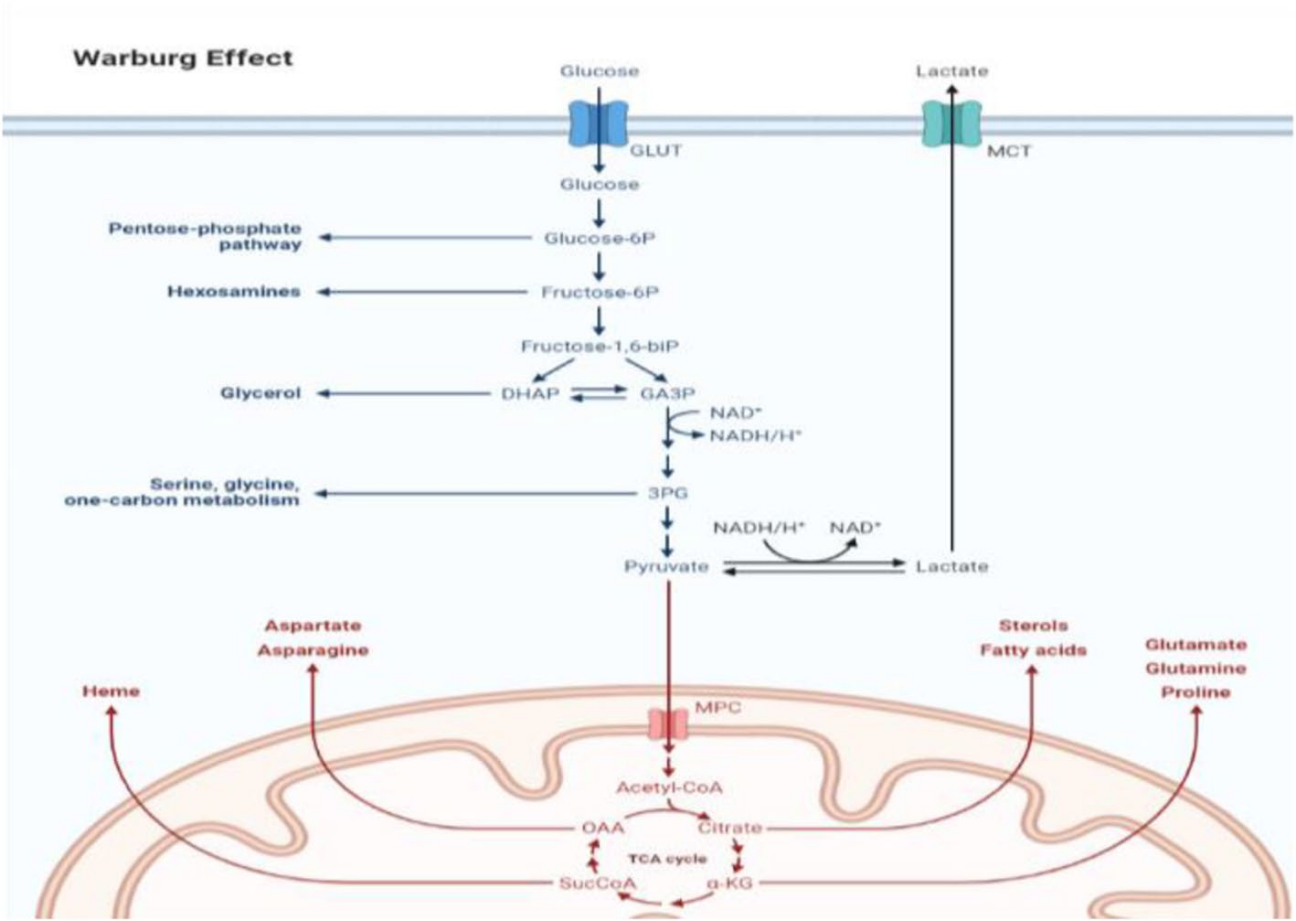
Schematic representation of Warburg effect with most of the flux is channeled toward lactate synthesis. In addition, note that in the case of cancer cells the glucose uptake rate is higher than that observed in the normal cells

**Fig. 8:**
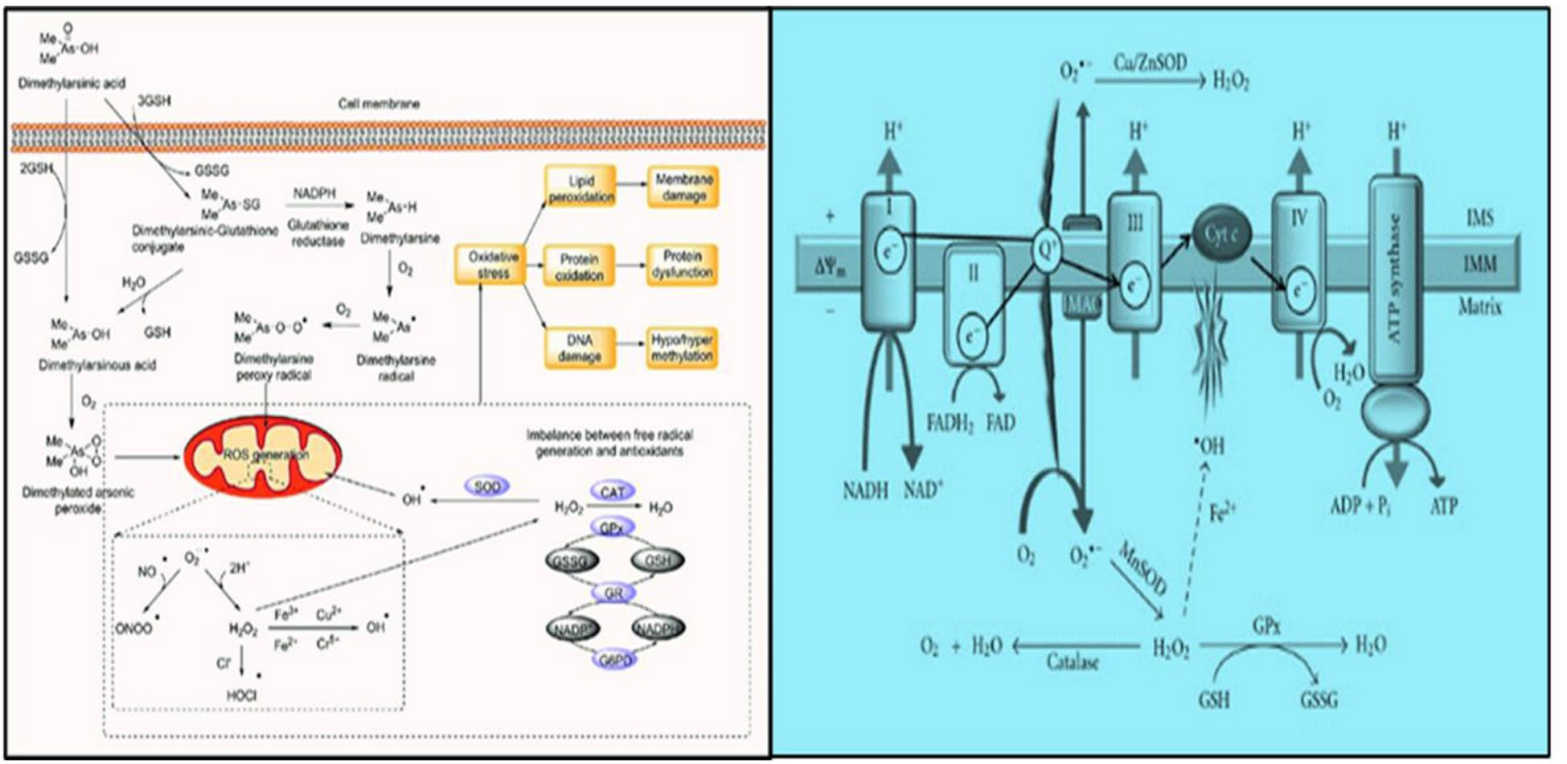
Oxidative stress by free radicals are depicted in this graphic as a result of SDH deficiency [84]

Superoxide and hydrogen peroxide are examples of ROS, which are harmful molecules containing oxygen with an unpaired free electron. Although ROS are necessary for normal cell activity, ROS may also affect DNA oxidation resulting in genomic instability and, apoptosis. Mitochondria are the primary generator of ROS. Complexes I and III can create a large number of ROS; however, CII can also produce a considerable amount of ROS. Succinate accumulation causes an over-reduced ubiquinone pool, which causes reverse electron transfer to complex I, where electrons escape as ROS. High amounts of ROS have been found to activate HIF and the pseudohypoxia pathway. SDHx mutation-induced increases in ROS have been demonstrated to promote genomic instability, which, in addition to HIF stabilization may share in carcinogenesis. Kluckova et al*.* developed different models of SDH failure with conflicting findings concerning ROS generation [36]**.** Abnormalities in tumor cell metabolism are a fundamental component of cancer compared to non-malignant cells [37]. Under the normoxic condition of normal cells, glucose is converted to pyruvate through the glycolytic pathway and then oxidized to CO_2_ in mitochondria. This metabolic process produces 36 molecules of ATP per molecule of glucose [38]. In the case of hypoxia, mitochondrial OXPHOS is inhibited, causing pyruvate conversion into lactate in a process known as aerobic glycolysis [39]. Moreover, glycolysis only produces 2 ATP per glucose molecule (Fig. 7). Otto Warburg was the first to identify metabolic reprogramming in cancer cells nearly a century ago [40] who observed that in comparison to non-malignant tissue, cancer cells acquire massive amounts of glucose. In cancer cells, there has also been a reduction in cancer cell respiration and an increase in glucose fermentation into lactate [41]. HIF-1 functions as a major regulator of glucose metabolism and cell proliferation, and hence plays an important part in the regulation of alterations leading to the Warburg phenotype [42]. As previously stated, The Warburg effect is seen in SDH-related malignancies and its effect is mediated by HIF, which increases the expression of glucose transporter 1 (GLUT1) and glucose transporter 3 (GLUT3), as well as hexokinase 2, pyruvate kinase M2 (PKM2), and lactate dehydrogenase A. (LDH-A).PKM2 interacts with HIF1 in the nucleus, it serves as a co-activator to facilitate the switch from oxidative to glycolytic phosphorylation by increasing the expression of HIF1 target genes In vivo, LDH enzyme converts pyruvate to lactate. Lactate production results in an acidic tumor microenvironment, which promotes tumor migration and metastasis and is associated with a poor prognosis [43]**.** Oxidative stress is the striking outcome of SDH dysfunction, the reduction of oxygen generates several free radical precursors, including superoxide radicals, hydrogen peroxide, and hydroxide radicals [44]**.** Many physiological functions are regulated by another ROS, nitric oxide (NO). The Fenton reaction produces the hydroxyl radical in the presence of Fe^+3^ when superoxide reacts with NO leading to the formation of peroxynitrite, a powerful radical. Other enzymes can produce ROS, including NADPH oxidases, xanthine oxidases, cyclooxygenases, and lipoxygenases. Several enzymes can degrade or inactivate ROS, including Superoxide dismutase, glutathione peroxidases, peroxiredoxin, and thioredoxin are all antioxidant enzymes. To maintain homeostasis, there is a delicate balance between the roles of ROS and antioxidants during development, with disturbance resulting in oxidative stress [45]. One of the two major mechanisms by which oxidative stress contributes to disease by causing aberrant cell function and death is the production of reactive species, particularly •OH, ONOO, and HOCl-, which directly oxidize cellular components, Fig. 8 [46].

## Treatment

### Chemotherapy

#### 5-Fluorouracil

5-fluorouracil (5-FLU) is a type of chemotherapy that is commonly used to treat a variety of cancers such as colorectal, breast, pancreatic, and lung cancers by disrupting uracil metabolism in cancer cells and slowing cancer progression [47, 48]**.**

**Fig. 9:**
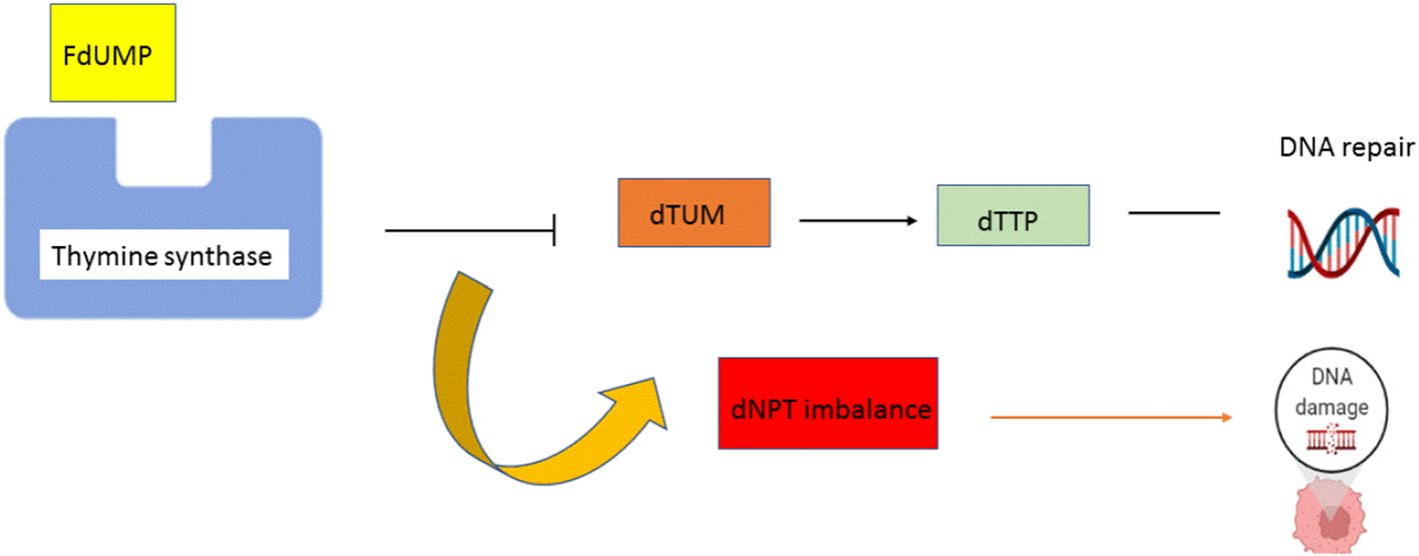
5-FLU mechanisms of action that depend on thymidylate synthase (TS) which catalyzes the conversion of deoxyuridine monophosphate (dUMP) to deoxythymidine monophosphate (dTMP)

Using 5,10-methylenetetrahydrofolate (CH_2_THF) as a methyl donor, thymidylate synthase (TS) catalyzes the conversion of deoxyuridine monophosphate (dUMP) to deoxythymidine monophosphate (dTMP). Fluorodeoxyuridine monophosphate (FdUMP), a 5-FLU active metabolite, binds to the nucleotide-binding site of TS and forms a stable ternary complex with TS and CH_2_THF, preventing dUMP access to the nucleotide-binding site and suppressing dTMP formation. Both dUTP and the 5-FLU metabolite, FdUTP, can be misincorporated into DNA, resulting in deoxynucleotide (dNTP) pool imbalances. In the presence of high dUTP/dTTP ratios, repair of uracil and 5-FLU-containing DNA by the nucleotide excision repair enzyme, uracil–DNA–glycosylase, is ineffective and only leads to more false-nucleotide incorporation resulting in further DNA damage and cell death (Fig. 9)[49].

#### Complications of 5-fluorouracil

Patients who receive chemotherapy and/or radiotherapy have improved surgical results; however, the impact on long-term survival has been negligible due to the extreme resistance of lung and pancreatic cancers to all currently available treatments. Chemotherapy with 5-FLU or radiotherapy provides a slight increase in survival in these individuals [50]. In contrast, the standard of care with modified FOLFIRINOX (with a modification to eliminate bolus doses of 5-FLU) has offered the longest median survival for those rare patients (10–15%) who are appropriate for surgical resection (54 months) [51]. The majority of these patients, however, relapse and die. As with several other malignancies, pancreatic cancer cells are frequently observed to have an excessive buildup of ROS. This finding contributes to the rationale for treating cancers with medications that raise ROS levels to a point, where they promote cell death; however, it may be more effective to promote cell death by increasing ROS generation while inhibiting antioxidant systems. Among the different emerging strategies for cancer therapy, targeting tumor bioenergetics may allow modulation of biochemical pathways involved in energy metabolism underlie tumor initiation, survival, and resistance to therapy. Combining these two approaches with natural treatments may represent a promising therapeutic option in a range of cancer types [52]**.**

### Phytochemicals treatment

Phytochemicals are natural substances that are secondary metabolites of therapeutic plants. Phytochemicals have anti-inflammatory and anti-cancer properties (through cell cycle participation, apoptosis, angiogenesis, and metastasis), and may also increase immunity and have antioxidant properties [53]. Multiple studies have reported the use of phytochemicals as chemo-preventive and chemotherapeutic agents in a variety of cancers [54]. According to the WHO, herbs represent the best source for a variety of medicines. Phytochemicals have promise as novel therapeutic agents in cancer as they have utility in targeting several cancer cell pathways and have low toxicity in normal cells [55]. Among phytochemicals are flavonoids that are present in a range of fruits, vegetables, and drinks, such as tea, chocolate, and wine [56]. In a previous study, it was found that the dietary flavonoids apigenin, chrysin, and quercetin which are present in grape and apple extracts, can block the activity of the proteasome in human cancer cells and cause apoptotic cell death [57]. The goal of the current research is to identify dietary flavonoids that are more strong and effective tumor inhibitors.

#### Chrysin

Chrysin (5,7-dihydroxy-2-phenyl-4H-chromen-4-one) is a natural flavonoid found in a range of plants, particularly chamomile, Pleurotus ostreatus, and honeycomb [55]. The anti-cancer and cancer-prevention properties of chrysin are notable among its biological features. The anti-cancer effects of chrysin are mediated by inhibiting and preventing a range of processes in cancer cells, including apoptosis, autophagy, angiogenesis, and cell proliferation. In general, the presence of a 5-hydroxyl group and C2–C3 double bond appears to be important for potent flavonoid P-glycoprotein are appears to be responsible for drug resistance [58]. Chrysin has a higher cytotoxic effect on cancer cells than on normal cells. The potential impacts of apoptosis on cancer cells from the breast, prostate, thyroid, pancreas, NSCLC, and gut have been thoroughly studied. Chrysin has been studied in vivo and in vitro for its anti-cancer properties including a range of cell lines, such as T47D, SW480, PC3, SKOV-3, and MCF-7 [59]. Chrysin is also more concentrated in the T47D cell line due to its molecular structure. A natural product ingredient called chrysin exhibits anticancer properties in a variety of cancer cell lines [60]**.** Chrysin has been the subject of numerous research that have attempted to explain the mechanisms underlying its anti-cancer properties, including suppression of the PI3K/Akt pathway, activation of caspase-3 and caspase-8 [61], and depletion of cellular glutathione [62]**.** Chrysin, an HDAC inhibitor, induces p21 to arrest the cell cycle and subsequently muzzles cellular growth. In addition, the aromatase inhibitor chrysin may slow the growth of tumors in breast cancers that express the oestrogen receptor-positive breast cancer MCF-7 Cells [63]**.** The biological anti-cancer capabilities of chrysin are now being investigated. Isolated mitochondria from B-lymphocytes isolated from patients with CLL have been used to demonstrate chrysin inhibits SDH activity in CLL cells without affecting mitochondria in normal cells. Furthermore, it was discovered that chrysin promotes increased ROS production linked with apoptosis in CLL B-lymphocytes via inhibiting CII [33]. CCNPs improved the inhibitory effect of chrysin on Succinate–coenzyme Q oxidoreductase expression and activity [64]. Chrysin, a plant flavone with HDAC inhibitory effects, is gaining popularity as a promising anti-cancer drug and has been shown to have cytotoxic effects in many cancer types [65]. In addition, we observed chrysin decreased tumor cell HIF-1α expression in vitro and inhibited tumor cell-induced angiogenesis in vivo [66, 67]. Chrysin may represent an effective inhibitor of angiogenesis and carcinogenesis as HIF-1α regulates not only VEGF but also many other cancer-related genes [64, 66]**.**

Phytochemicals such as chrysin have various drawbacks and restrictions that impede their biological function; however, improvements in nanotechnology have considerably mitigated these drawbacks. Short half-life, limited solubility, poor biological availability, short circulatory stability length, and fast metabolism and degradation are only a few of these drawbacks [68].

### Nano-formulation technique

Nano-formulation is a nanometer-scale technology whose functional qualities are determined by factors other than its size. Scientists have devoted considerable attention to the application of this technology for the controlled transmission, discharge, and release of pharmaceuticals, particularly in cancer treatment [69]. Natural nanoscale compounds are showing promise as a strategy. The solubility may be improved, because the nanoscale alteration raises the surface area to volume ratio [70]**.** Small molecules are assembled into a nanoscale structure using a manufacturing process (such as organic synthesis and self-assembly on proteins) [71]. Chemical conjugation with other substances was done to enhance chrysin's functions. Chrysin's anti-inflammatory action was elevated over that of chrysin alone when it was conjugated with indole and barbituric acid [72]. Chrysin-like antioxidative activity was present in organogermanium [73]. And through apoptosis-associated mitochondrial function and anti-angiogenesis, conjugation of chrysin-organogermanium produced synergistic antioxidant benefits and improved anticancer effects [74]. Chrysin with selenium demonstrated considerable cytotoxicity with an IC_50_ that was 18-fold lower than that of chrysin. In addition, cisplatin, a widely used anticancer medication, could not compete with the deadly action of selenium-containing chrysin [75]. These findings imply that conjugation increases the therapeutic efficacy of compounds such as chrysin and others with comparable biological action.

Another strategy for overcoming chrysin's drawbacks is to use a drug delivery system (DDS). Drugs can have their characteristics changed or made more soluble via DDS. DDS could also be used to build up encapsulated medication at a particular spot (e.g., tumor) [76]**.** A variety of materials have been used to prepare drug-containing nanocarriers, including natural and synthetic polymers, lipids, and surfactants. Among the materials proposed for mucosal delivery, polysaccharides have received considerable attention due to their outstanding physical and biological properties [77]. Chitosan (Cs) is a biopolymer made up of 1–4 connected 2-amino-2-deoxy-glucopyranose (GlcN) and 2-acetamido-2-deoxy-d-glucopyranose (GlcNAc). Cs is a polysaccharide made from chitin that has undergone deacetylation. Chitin is freely available and naturally occurring as it is found in the structural components of many invertebrates, as well as the cell walls of most fungi and algae. Due to its chemical, physical, and functional properties, Cs is also regarded as a highly versatile polymer. These benefits include its cationic nature, biodegradability, high adsorption capacity, biocompatibility, permeability-enhancing effect, film-forming capabilities, and adhesive features. Furthermore, Cs is regarded as safe and cost-effective [78]. We recently demonstrated chrysin and CCNPs are more potent inhibitors of the SDH and ubiquinone oxidoreductase activities of CΙΙ, respectively. This has previously been proven theoretically using molecular docking [64].

**Fig. 10:**
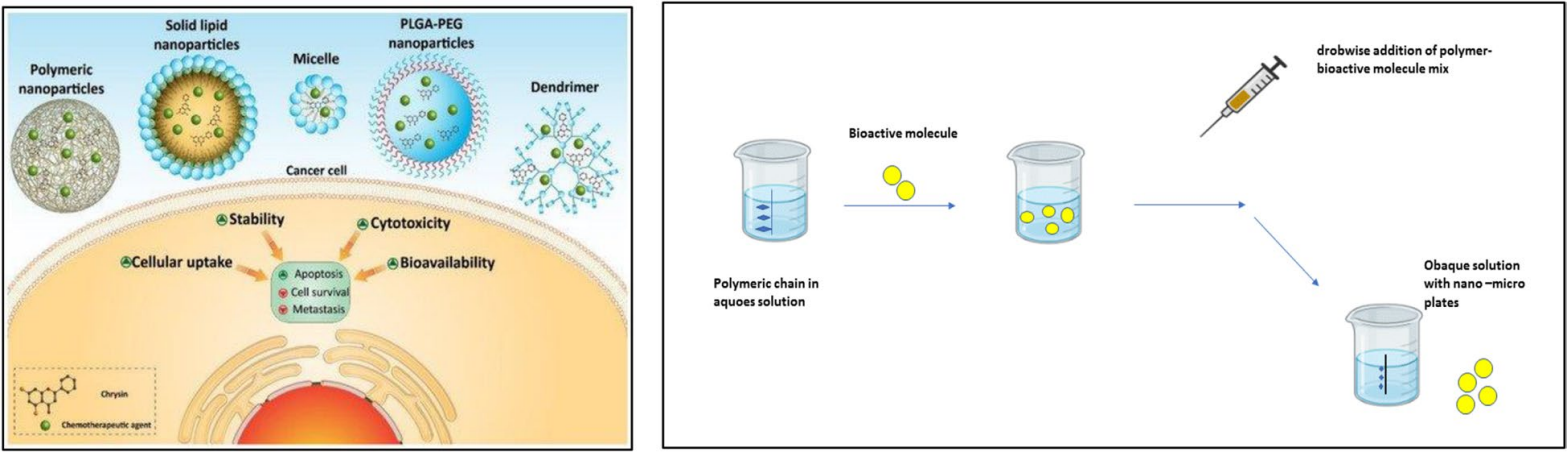
Chrysin-loaded nanoparticles in cancer therapy [80]. The ionotropic gelation process is used to synthesize nanoparticles and microparticles, as shown in this diagram. The procedure is carried out with constant stirring and at a temperature of roughly 25 °C. Centrifugation can be used to collect particles in a dispersion

Cs is more biocompatible than other polysaccharides and can be altered through ionic cross-linking and covalent binding to create a variety of nanomaterials, including nanoparticles, films, nanogels, and fibres [79, 80]**.** The ionic gelation method for bioactive encapsulation involves the creation of multiple electrostatic contacts between oppositely charged polymers, polycationic Cs, and a polyanion. Sodium tripolyphosphate is commonly used as the basis of the ionic gelation process (TPP). Chitosan nanoparticles have predominantly been used in the pharmaceutical industry as carrier materials or as a delivery method for proteins, medicines, vaccines, and/or DNA (Fig. 10) [78].

## Conclusions

Mitochondrial complex ΙΙ (CΙΙ) functions in oxygen sensing in addition to mitochondrial energy generation. CII plays a central role in the Krebs cycle and respiratory chain and is also associated with SDH-related human diseases. We predict chrysin and CCNPs to have considerable therapeutic effects on both functions of mitochondrial CΙΙ in cancer cells owing to their inhibition of mitochondrial CΙΙ and ROS generation, thereby inducing apoptosis through suppression of ATP generation in cancer cells.

## Data Availability

Not applicable.
